# Supplementary material for: Epidemiological characteristics of ischemic heart disease: A comparative study between China and the world from 1990 to 2021 and prediction to 2036
Source: PLoS One. 2025 Dec 19;20(12):e0336963. doi: 10.1371/journal.pone.0336963 (PMC12716773; doi:10.1371/journal.pone.0336963)
Supplement: Attachment — Submitted filename: PONE_D25_41345_China_IschemicHeartDisease.docx [file pone.0336963.s007.docx]

Plos-One Review

Key Word: China. IHP Cases, Community Health Data analysis.

Brian L Altonen

PONE-D-25-41345.

Jiale Wu, Jinchan Zhai, Xingyun Zhu, Quan Hao, Jiaqi Yang, Xiaoju Li, Yunhua Hu, Qiang Niu. Yizhong Yan. Epidemiological characteristics of ischemic heart disease: a comparative study between China and the world from 1990 to 2021 and prediction to 2036.

**Article**

This is a retrospective community health data study that evaluates data contained in a database containing global health data related to numerous countries.

The country/region that serves as the center for this project, and the placement of its researchers, is China. The researchers also made use of the University of Washington research programs which are in general devoted to studies focused on Pacific Rim population health and health economy topics.

The data were evaluated for this article by researchers using Joinpoint regression model method developed for this type of projection and disease prediction study used to evaluate the burden of covariates included in this work. The database itself used for the original data is Global Burden of Disease [GBD] Study, consisting of data for the period 1990 to 2021, published by the Center for Health Measurement and Evaluation, University of Washington.

The data elements (specific variables) reviewed for this temporal change or comparison study are age-standardized incidence rate (ASIR), age-standardized prevalence rate (ASPR), and age-standardized mortality rate (ASMR). The modeling formula used was referred to by the writers as the Bayesian Age-Period-Cohort Model. Primary data comparison outcomes are reported as percent values and standardized rates reporting (i.e. #/100,000).

The addition of other methods for data modeling such as use of nested Laplacian approximate BAPC method and the Integrated Nested Laplace Approximation (INLA) method may help with further advancement in these tasks in the future.

Through the reviews of changes over time, prediction models can be developed, the theoretical accuracy of which is derived by relating the best formulas use to produce those graphed relationships, with least variability, greater and more consistent, repeatable range p values.

Cross comparisons with “other datasets” Other parts of the world, other country types. See Section 3 ending.

**Presentation/Writing Style**

Grammer -- Narrative; nice to read, but sometimes approaching semi-formal.

Spelling -- one misspell in section title. See Line 179: “3.3 Comarison” (sic)

**Figures/Tables/Etc.**

ASMR, ASPR, ASIR Prediction drawings – quite interesting

IHD Cases (y), yearly x-axis -nice

Incid, Prev., Mortality Tables - useful

**Review, Comments**

**Innovation Scale 3.5**

Levels

Summary/repeat [1], has newness [2], **novelty [3], very important [4],** innovative [5]

China has a reasonable academic, professional population of science researchers. This adds to the knowledge base, and has broad applications. But in a sense it “feels” fairly typical or “common” for the kind of work. Similar projects are expected in developed countries. The importance of this work pertains to the need for it to be managed more completely by experts in this particular academic community. This type of work can and should be done in a cross-comparative style (outcomes differences within the country’s health care system and population).

A more complete, and full application of this research-analysis method will provide important insights.

**Summary**

Publish. No serious or sizeable text changes.
