# Supplementary material for: Epidemiological characteristics of ischemic heart disease: A comparative study between China and the world from 1990 to 2021 and prediction to 2036
Source: PLoS One. 2025 Dec 19;20(12):e0336963. doi: 10.1371/journal.pone.0336963 (PMC12716773; doi:10.1371/journal.pone.0336963)
Supplement: Attachment — Submitted filename: Response to Reviewers.docx [file pone.0336963.s009.docx]

Response to Reviewers

We would like to express our sincere gratitude to all the reviewers for their valuable comments and constructive suggestions, which have been of great help in improving our manuscript. We have carefully addressed each comment, and the detailed responses are as follows:

**Response to Reviewer #1**

Thank you for your positive comment on our study regarding the prevalence and incidence of ischemic heart disease (IHD) in specific populations. We are pleased that our research has highlighted the complexity of this disease and the roles of lifestyle and genetics, as well as the high incidence in China's large population, which indeed underscores the need for more intervention measures. We will continue to pay attention to this issue and explore more effective strategies in future research.

**Response to Reviewer #2**

We are grateful for your comprehensive and insightful comments, as well as your positive evaluation of the manuscript's detail and writing. We have carefully revised the manuscript according to your suggestions:

1.Regarding the data source section: We have added a brief description of the sample size and data collection methods of GBD 2021 in the revised manuscript. Specifically, GBD 2021 provided epidemiological data for 204 countries or regions, 371 diseases, and 88 risk factors. This addition is intended to help assess the generalizability of the study.

2.Regarding ethical guidelines: The data for this study were sourced from a publicly available database, requiring no ethical approval or informed consent. No ethical approval and informed consent were required because of the public availability of GBD and no identifiable information was included in the analyses.

3.Regarding the data analysis section: We have more accurately described how the joinpoint and Bayesian age-period-cohort (BAPC) models were used to synthesize data from 1990 to 2021, and have also added explanations of key parameters such as the initial joinpoint and the number of joinpoints to provide more detailed information.

4.Regarding tables and confidence intervals: We have checked all tables (including Table 3) and explicitly indicated statistical significance in the text, with all relevant values marked with significance symbols. We have also ensured that confidence intervals (e.g., (3.67, 3.92)) are correctly displayed throughout the manuscript through careful editing. Additionally, we have revised the manuscript to ensure that the key points can still be conveyed through the text even if the tables are not visible.

5.Regarding the discussion section: We have strengthened the discussion by adding additional evidence and appropriate references to support our interpretations and claims.

**Response to Reviewer #3**

Thank you for pointing out the spelling error in the chapter title, which we have corrected in the revised manuscript. We also appreciate your comment on the writing style; we have reviewed the text to make it more formal while maintaining its structural clarity. We agree with your view that the review of new databases is crucial for the field, especially in the context of changes in artificial intelligence, data analysis, and management services in healthcare. We will continue to contribute to the advancement of research in this area.

Once again, we would like to thank all the reviewers for their time and efforts in reviewing our manuscript. We hope that the revised version meets your expectations.
